# Developing a Questionnaire Evaluating Knowledge, Attitudes and Behaviors on Audit & Feedback among General Practitioners: A Mixed Methods Study

**DOI:** 10.3390/healthcare11091211

**Published:** 2023-04-24

**Authors:** Angelo Nardi, Suzanna Mitrova, Laura Angelici, Camillo Giulio De Gregorio, Donatella Biliotti, Corrado De Vito, Simona Vecchi, Marina Davoli, Nera Agabiti, Anna Acampora

**Affiliations:** 1 Local Health District 2, Local Health Authority Roma 1, 00193 Rome, Italy; 2 Department of Epidemiology of the Regional Health Service of the Lazio Region, Local Health Authority Roma 1, Via Cristoforo Colombo, 112, 00154 Rome, Italy; 3 Local Health District 13, Local Health Authority Roma 1, 00193 Rome, Italy; 4 Department of Public Health and Infectious Diseases, Sapienza University of Rome, Piazzale Aldo Moro, 5, 00185 Rome, Italy

**Keywords:** audit, feedback, general practice, survey, knowledge, attitudes, behaviors

## Abstract

Background: Audit and Feedback (A&F) is one of the most common strategies used to improve quality in healthcare. However, there is still lack of awareness regarding the enabling factors and barriers that could influence its effectiveness. The aim of this study was to develop a questionnaire to measure the knowledge, attitudes and behaviors of general practitioners (GPs) regarding A&F. The study was performed in the context of the EASY-NET program (project code NET-2016-02364191). Methods: The survey was developed according to two steps. Firstly, a scoping review was performed in order to map the literature on the existing similar instruments with the aim of identifying the sub-domains and possible items to include in a preliminary version of the questionnaire. In the second phase, the questionnaire was reviewed by a multidisciplinary group of experts and administrated to a convenience sample in a pilot survey. Results: Ten papers were included in the scoping review. The survey target and development methodology were heterogenous among the studies. The knowledge, attitudes and behaviors domains were assessed in six, nine and seven studies, respectively. In the first step, 126 pertinent items were extracted and categorized as follows: 8 investigated knowledge, 93 investigated attitudes, and 25 investigated behaviors. Then, 2 sub-domains were identified for knowledge, 14 for attitudes and 7 for behavior. Based on these results, a first version of the survey was developed via consensus among two authors and then revised by the multidisciplinary group of experts in the field of A&F. The final version of the survey included 36 items: 8 in the knowledge domain, 19 in the attitudes domain and 9 in the behaviors domain. The results of the pilot study among 15 GPs suggested a good acceptability and item relevance and accuracy, with positive answers totaling 100% and 93.3% in the proposed questions. Conclusions: The methodology used has shown to be a good strategy for the development of the survey. The survey will be administrated before and after the implementation of an A&F intervention to assess both baseline characteristics and changes after the intervention.

## 1. Introduction

Audit & Feedback (A&F) is one of the most common strategies used to improve quality in healthcare and can be defined as “any summary of clinical performance of healthcare over a specified period of time aimed at providing information to health professionals to allow them to assess and adjust their performance”. During an audit, a systematic review of professional performance is performed based on explicit criteria or standards. The results of the review are then fed back to health professionals in a structured manner [1]. The most recent Cochrane meta-analyses confirmed that A&F interventions can improve clinical practice [2]. It was found that the impact of these interventions ranged from small to moderate in terms of enhancing compliance with the desired practice, with a moderate grade of evidence. Due to heterogeneity in the effectiveness of the included studies, the authors attempted to elucidate which factors could be associated with better outcomes, suggesting that they might depend on both the feedback and contextual characteristics. However, indirect comparisons and the reporting quality of the primary studies [3] limit the production of general assumptions. Furthermore, in a recent update, it was found that new studies comparing A&F to usual care added little new insight when aiming to better understand which factors are more relevant than others [4]; this suggests the need to perform studies that are aimed at exploring the enabling factors and barriers of A&F [5].

To understand how A&F works, researchers have recently focused on both individual [6] and contextual factors [7], as well as on intervention characteristics [8], which can influence the effectiveness of A&F strategies. Following these indications, 15 recommendations to develop effective feedback were identified [9], and different approaches were used to elucidate contextual [7] and individual [6] factors, including physicians’ attitudes and beliefs. In the study by Hut-Mossel, a context–mechanism–outcome configurations approach was proposed in order to investigate the link between contextual, individual, and intervention characteristics in determining the outcomes. According to this model, physicians’ beliefs, attitudes, and logic, together with contextual factors, play a role in determining behaviors. In Desveaux’s study, the authors concluded that intervention characteristics are not sufficient in order to explain A&F efficacy, suggesting that evaluating physician and contextual factors may be important when a new A&F strategy is implemented. From these studies, the impact that peculiar clinical and organizational settings may have on the effectiveness of A&F interventions was determined.

A&F has been widely used in hospital settings, but various examples of different forms of A&F can also be found in primary care [10]. In primary care, studies have been focused mainly on the quality of care for patients affected by chronic diseases [11,12] and on the physician’s prescribing behaviors [13,14]. Improvements were evaluated using both process and outcome indicators, with the former achieving better results. Although most of these interventions were generally aimed at enhancing the use of best practices and clinical guidelines, it is well known that in general practice, specific contextual characteristics, such as multimorbidity [15] and pharmacological interactions [16], can hinder the use of clinical guidelines. In a recent work, Jamtvedt et al. synthesized A&F interventions implemented at both national and local levels in European countries [17]. At a national level, the Quality and Outcome Framework, a pay-for-performance scheme introduced in the United Kingdom in 2004 by the National Health Service, adopted a set of indicators developed by the National Institute for Health and Care Excellence, which aims to increase the use of evidence-based practices and to reduce the variability in the performance of general practitioners (GPs). In the Netherlands, clinical and organizational audit is part of the accreditation process for primary care practices, and specific pharmacotherapy audits are routinely used by almost all GPs. A yearly quality strategy that uses, among others, chronic care indicators on diabetes and cardiovascular diseases, is delivered to one-fifth of all of Finland’s health centers.

The present study was performed in the context of the EASY-NET program, “Effectiveness of Audit & Feedback strategies to improve healthcare practice and equity in various clinical and organizational setting” (project code NET-2016-02364191), which was co-founded by the Italian Ministry of Health and by participating Italian regions. It aimed to compare the efficacy of different A&F interventions in various clinical and organizational settings, in order to also understand possible enabling factors and barriers. 

In particular, the Lazio Region was involved in work package 1, which experimented with an A&F intervention that involved healthcare professionals from local health authorities and GPs with the aim of improving clinical and organizational practice in the context of chronic care pathways for patients affected by diabetes and chronic obstructive pulmonary disease (COPD). The main recipients of the intervention were GPs. They were invited to participate in a scheduled audit meeting that also involved specialists (pneumologists or diabetologists) and professionals responsible for the management of health services and for systematically reviewing the practice. The results of the selected quality indicators, such as the proportion of diabetic patients with at least one glycated hemoglobin test in a year, adherence to the treatment of COPD patients with bronchodilators, flu vaccination coverage and hospitalization rates, were then fed back to GPs and actions for improvement were identified in subsequent meetings. Within the program, one of the research activities was to evaluate the efficacy of the implemented intervention in terms of improving the knowledge, attitudes, and behaviors of the participating GPs regarding A&F. In this regard, the present study aimed to develop a questionnaire to evaluate the knowledge, attitudes, and behaviors of GPs regarding A&F by performing a scoping review, an informal consultation with experts in the field and a pilot study.

## 2. Materials and Methods

The survey was developed according to two steps. Firstly, a scoping review was performed to map the literature on the existing similar instruments in order to identify concepts and possible items to include in the present questionnaire. In the second phase, the preliminary version of the questionnaire was reviewed by a multidisciplinary group of experts and administrated to a convenience sample of GPs in a pilot study.

### 2.1. Scoping Review

#### 2.1.1. Search Strategy, Information Sources and Eligibility Criteria

A search string was built by two researchers with the support of a documentalist and run in November 2021 through the MEDLINE, Embase, and PsycINFO databases. The search string was adapted to the specific options of each database (Supplementary Materials). English language surveys that measured the knowledge, attitudes, and/or behaviors of healthcare professionals regarding A&F were included. All healthcare professionals were considered. To be included in the review, papers had to focus on the knowledge, attitudes, or behaviors of health practitioners regarding A&F, and report the items used to assess at least one of these domains. Alternatively, the items had to be inferred from the article. The instruments used for the assessment in the included studies could be validated or not. Studies were excluded if the target was represented by patients or health managers and if it was not possible to extract items that were consistent with the aim of the study. Attitudes towards giving feedback, peer feedback strategies, and assessment regarding the implementation of a specific audit strategy that cannot be applied to A&F in general were not considered.

#### 2.1.2. Data Charting Process and Synthesis of Results

The *Rayyan*© [18] web-tool was used to remove duplicates and to facilitate the screening process. After screening the three databases, a snowball search was performed from the included studies in order to detect other relevant studies.

Data were extracted by one researcher and doubts were solved via discussion with another researcher. A standardized form was used to extract data that were relevant to the study aim. The data extracted were as follows: authors, year of publication, survey target (in terms of healthcare professionals and specialty), survey items with the corresponding domain (knowledge, attitudes or behaviors), and measurement, such as rating scale (i.e., Likert scale, frequency scale) or dichotomous answer (yes/no). 

### 2.2. Survey Development and Pilot Study

After data extraction, survey development followed three additional steps: the identification of the preliminary items, the experts’ review, and the definition of the final items.

In the preliminary items’ identification step, for each domain, the extracted items were categorized into different sub-domains depending on the underlying concept. Similar items or items investigating the same concept were combined and a single question was formulated. Two researchers discussed the list of items generated and modified or eliminated items that were considered not pertinent to the study aim. The final decision was reached via consensus with a third author. At the end of the first step, each item was converted into a question and the possible responses were defined as appropriate. 

In the second step, the first version of the survey was revised by a multidisciplinary group of six experts in the field of A&F (two experts in clinical audit, two experts in feedback elaborations, and two representatives of health services management). The preliminary questionnaire was sent to the experts via e-mail along with the instructions to follow in order to give their opinion. They were asked to indicate whether an item should be removed, retained or modified. If they indicated that an item should be modified, the experts were asked to modify the item using the track changes function in Word©. At the end of the review process, decisions were made according to a majority criterion. Items were excluded if at least four experts (67%) indicated that it should be removed. If modification was required, the suggestions were collected and discussed among three researchers and the final decisions were made via consensus. An updated version of the questionnaire was then sent via e-mail to the same group of experts, additional open comments were collected and, where needed, a final re-elaboration of the items was carried out. In this phase, the rating scale for each item was also defined. In general, except for dichotomous ones (Yes or Not), all the responses were collected according to a 5-point Likert scale, where 1 was the lowest score and 5 was the highest score. Finally, the questionnaire was transformed into an electronic form.

In the final step of the questionnaire elaboration, a pilot study was performed in order to evaluate the acceptability, relevance of the items, and the accuracy of the questionnaire. For this purpose, four additional questions were included at the end of the questionnaire. The participants could express their evaluation of the first four questions according to a 5-point Likert scale.

- In your opinion, how easy was the questionnaire to answer?
- In your opinion, how do you rate the readability of the questions?
- In your opinion, how do you score the relevance of the included items?
- In your opinion, how accurate is the questionnaire?

A convenience sample of GPs not participating in the EASY-NET project was recruited and the final version of the questionnaire was electronically delivered using Google Forms.

The responses were firstly categorized into either low scores, which ranged from 1 to 3, and high scores, which ranged from 4 to 5. These scores were then analyzed according to a majority criterion. If at least 51% of the respondents gave a specific aspect of the questionnaire a low score, this was revised with respect to the critical point highlighted (i.e., readability). If at least 51% of the respondents gave a specific characteristic of the questionnaire a high score, this aspect was confirmed.

## 3. Results

### 3.1. Scoping Review

#### 3.1.1. Selection of Sources of Evidence

**Figure 1:**
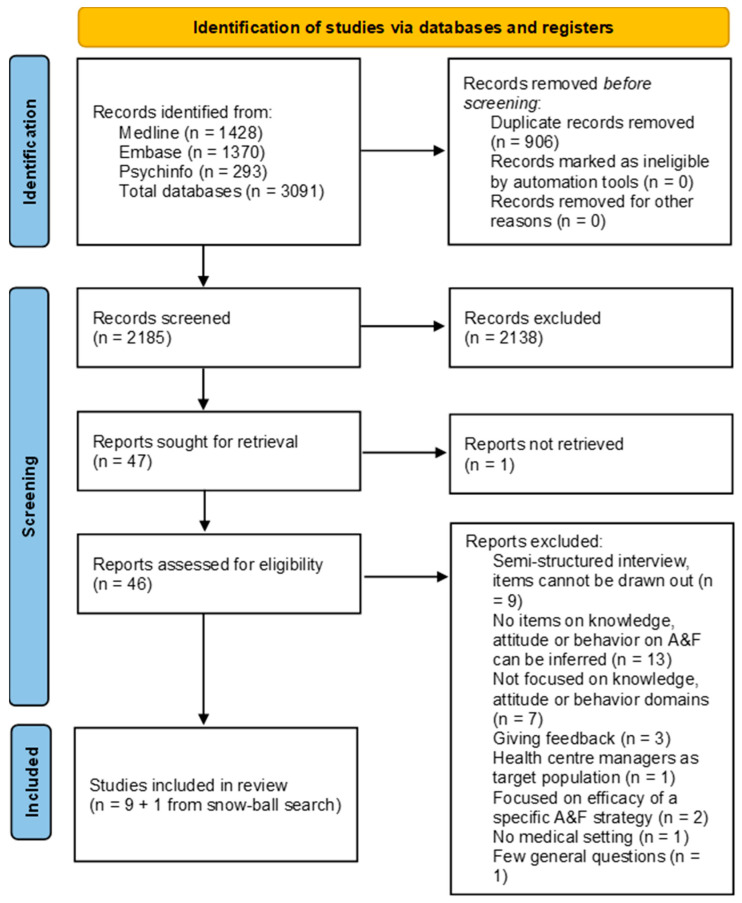
PRISMA 2020 flow diagram for new systematic reviews that included searches of databases and registers only.

The search string produced a total of 3091 results in the three databases. The whole screening process was summarized in the PRISMA Flow Chart (Figure 1) [19]. After removing duplicates, 2185 papers were screened. Of these, 2138 were excluded after title and abstract screening, and of the remaining 47, 37 were excluded after full-text screening. In total, 9 papers [20,21,22,23,24,25,26,27,28] met all the inclusion criteria. Furthermore, one additional study [29] was identified through a snowball search.

Exclusion was mainly due to the absence of items that evaluated at least one of the domains of knowledge, attitudes, or behavior, or because semi-structured interviews were used and items could not be drawn out. In one of the included studies [23], a scale developed to assess attitudes toward standardized assessment scales was used. The study was included in the scoping review as standardized assessment was considered a key component of feedback strategies. One study was identified through a snowball search. Three studies that used the same surveys adopted in the included papers were not considered because the aim of the snowball search was to detect additional surveys or items concerning A&F. 

#### 3.1.2. Characteristics of Source of Evidence and Synthesis of Results

The main characteristics of the 10 included studies are reported in Table 1. The included studies were published between 2012 and 2021. Four studies were conducted in Europe [24,25,26,29], three were conducted in the USA [20,22,23], and one was conducted in each of Asia [28], Canada [27], and Australia [21]. The survey target was represented in four studies that focused on trainee physicians: trainee general practitioners [28]; foundation year one doctors [29]; residents of any specialty [22], and psychiatry [24]. The targets of the other studies were physicians of various specialties [26], multidisciplinary mental health teams [23], multidisciplinary oncology teams [21,25], and surgeons [20,27].

The knowledge domain was assessed in six studies [20,21,25,27,28,29], the attitudes domain in nine studies [20,21,23,24,25,26,27,28,29], and the behaviors domain in seven studies [20,22,24,25,26,27,29].

Seven studies [20,21,23,25,27,28,29] used a five-point Likert scale, one study used questions with dichotomic answers [24], one study used open-ended questions [26], and one used a four-point frequency scale [22].

The studies showed heterogeneity in the development of the survey. Three of them were based on a preexisting theoretical framework [20,21,23]. In Stone [21] and Fahim [20], the survey items were then defined by the authors based on a specific methodology or on preestablished domains that had been identified by the theoretical framework. In Jansen-Doss [23], items were selected and adapted from preexisting measures and then modified, taking into account the suggestions that emerged from six pilot surveys. Ghaderi [27] conducted a literature review and proposed a set of items that was reviewed by a third author in order to assess the content validity. The final version of the survey was defined via consensus among the authors. In one study that used a semi-structured interview to assess knowledge and attitudes [26], the authors developed items in three thematic areas (general opinion about feedback, subjective impact of receiving feedback, and perception of the usefulness of pharmacists’ feedback in terms of preventing prescription errors). The survey used by McWilliams [24] was developed by taking into account themes that emerged from a focus group involving 14 general practitioners. In two studies [25,29], the authors stated that the questionnaire was developed using the existing literature on A&F. Two studies [22,28] did not mention the development of the methodology.

### 3.2. Survey Development and Pilot Study

In the first step (preliminary items identification), 169 items were extracted from the 10 included papers. Of the 169 items, 43 were excluded because they were considered not pertinent to the study aim. The remaining 126 items investigated the three domains of interest, as follows: 8 investigated knowledge, 93 investigated attitudes, and 25 investigated behaviors.

The extracted items were categorized, leading to the identification of 2 sub-domains for knowledge, 14 sub-domains for attitudes, and 7 sub-domains for behaviors. In particular, eight knowledge items were categorized into those investigated as “General knowledge on A&F” and those investigated as “Skills on A&F”. Regarding attitudes, items were found to investigate A&F in the following sub-domains: general attitudes regarding A&F; improving the quality of assistance (in terms of appropriateness, efficacy, organization, safety); improving assistance in different areas of medicine; long-term effectiveness; impact on modifying clinical practice; the validity of the information provided by the feedback; role of A&F in planning activities; attitudes towards the use of a benchmark; social norms regarding A&F; resources, costs and time used by A&F; and whether A&F ought to be mandatory. Behaviors items were classified into the following: previous experience; actual participation or willingness to participate in future A&F activities; behaviors modified after participation in A&F activities; frequency of report consultation; and peer consultation about the process and outcome indicators.

A first version of the survey was developed using the list of items retrieved via the scoping review, via consensus among two authors and via consultation with a third one. Where appropriate, an item was transformed in the corresponding question, while similar items were unified into a single question. The first version of the survey was then composed of 47 questions in which the knowledge domain included 11 questions, the attitudes domain included 20 questions, and the behaviors domain included 16 questions.

For most of the questions, the respondents could provide his/her answers according to a 5-point Likert scale, where 1 was the lowest score. Three questions required a dichotomous response of “Yes or No”.

In the second step, the first version was revised by a multidisciplinary group of experts in the field of A&F. One item in the knowledge domain (“Do you know the different steps of the clinical audit and feedback cycle?”) was eliminated, since four experts suggested that it should be eliminated because it was considered too specific. Another item in the attitudes domain (“Do you think that A&F activities could favor a specific healthcare aspect over all the others?”) was removed due to its lack of readability (4/6 experts indicated to remove it). Two experts suggested that the items in the attitudes domain be reformulated;, for example, “How important do you think A&F is useful to improve your clinical practice?” was reformulated to “Do you think A&F is useful to improve your clinical practice?”. The modification was then discussed among the researchers and applied. Finally, similar items were further aggregated in order to reduce the number of questions. The new version of the questionnaire was sent to the group of experts for final review. All the experts agreed with the proposed revision. No further comments were provided.

The final version of the survey was composed of 36 items: 8 knowledge, 19 attitudes, and 9 behaviors (Supplementary Materials).

A convenience sample of GPs was invited to participate in the pilot study and was asked to complete this version of the questionnaire. Out of the 35 invited, 15 GPs (42.9%) responded to the questionnaire. The retrieved results suggested the good acceptability, relevance, and accuracy of the questionnaire (Table 2). Regarding its acceptability, positive answers accounted for 93.3% and 100% (scored 4 or 5) of answers in regard to the questionnaire’s ease of answering and the perceived readability of the items, respectively. Positive answers accounted for 93.3% of answers regarding the testing relevance and accuracy of the questions. As the pilot study showed good results, the final version of the questionnaire was confirmed without further changes.

## 4. Discussion

This study aimed to develop a questionnaire to measure the knowledge, attitudes, and behaviors of GPs regarding A&F. The resulting survey was composed of 36 items: 8 knowledge, 19 attitudes, and 9 behaviors items. The methodology used to develop the survey followed two steps: first, a scoping review was conducted to identify items that could be potentially included in the survey; then, the items were categorized into sub-domains and selected by two authors.

The inclusion criteria of the scoping review were defined to be as comprehensive as possible in order to evaluate all the potentially suitable items. As a result, on one hand, the included instruments were heterogeneous in terms of the development methodology, target population, and purpose. On the other hand, the findings showed a lack of studies specifically focused on the development of surveys evaluating the knowledge, attitudes, and behaviors of GPs regarding A&F. One of the included studies [27] reported a methodology used to develop a questionnaire similar to the one adopted in the present work. The survey was addressed to surgeons. Regarding the target population, only one study assessed the knowledge and attitudes of GPs [28]. In particular, the target population of this study was trainee GPs that attended a yearly course on ‘‘Clinical Audit Skills’’ as a part of their training programme. In this survey, six questions were administrated only after the audit course. Four out of six questions concerned the knowledge domain and were used with the main purpose of evaluating the acquired competencies of GPs regarding A&F.

The scoping review showed that attitudes was the most frequently evaluated domain. It was assessed in all but one study, while behaviors and knowledge were assessed in seven and six out of ten studies, respectively. Furthermore, most of the items assessed in the included studies pertained to attitudes, with 93 items extracted. The items extracted in the behaviors and knowledge domains totaled 25 and 8, respectively. This proportion reflects the number of sub-domains identified by the present study: 14 sub-domains for attitudes, 7 sub-domains for behavior, and 2 sub-domains for knowledge. The higher number of items in attitudes compared to the knowledge domain was in part expected because of the exclusion criteria adopted in the scoping review. Indeed, studies that evaluated knowledge regarding A&F on a specific topic were not considered and only knowledge items that could be applied to every A&F intervention were included in the survey.

Concerning attitudes, it is noteworthy that there is consistency between the main issues regarding A&F barriers found in the literature and some of the sub-domains identified using the present methodology. In particular, the well-known need expressed by physicians to identify actions in practice that help to implement desired behaviors [6,30] can be considered captured by some of the sub-domains with a higher number of items; these include “improving quality of assistance” and “impact on modifying clinical practice”. Further, the “resources, costs and time” sub-domain investigated one of the most reported barriers to the implementation of A&F [31] and change in clinical behaviors in general [32].

Although the effectiveness of A&F interventions for GPs may be influenced by physicians’ individual factors, few studies evaluated the impact of knowledge and attitudes on the implementation of A&F strategies in general practice. Regarding feedback indicators, Foy et al. [33] found that certain domains of the Theoretical Framework Domains may impact on GPs’ adherence to evidence-based indicators. In particular, the “social and professional roles”, “identity and environmental context” and “resources” domains were found to influence all the indicators evaluated, while other domains, such as “beliefs about consequences”, “social influences” and “knowledge”, were found to possibly have a different impact depending on the indicator considered. More in general, knowledge and attitudes were found to be related to the use of evidence-based medicine by GPs [34]. In that regard, Cabana et al. [35] proposed a framework in which barriers to the implementation of clinical guidelines were grouped into knowledge, attitudes, or behavior domains. These findings confirmed the importance of assessing physicians’ knowledge and attitudes due to the fact that changes in these domains can be followed by changes in clinical practice [36].

The methodology used to develop this survey represents one of the strengths of the study. The scoping review allowed us to be comprehensive in terms of the items included in the survey. Another strength of the study is that survey was not specific to a particular intervention and thus can be applied to every A&F strategy in the context of general practice.

### Limitations and Future Research

The study also presents some limitations. A limit was represented by the nature of the survey, which is self-reported. Another limitation is that a structured validation of the questionnaire was not conducted. However, each item was selected and included in the survey after a consensus was achieved between two authors and confirmed by a third author. Items were, then, included after consensus among experts from different relevant disciplines (clinical audit, audit & feedback methodologies, epidemiology, and health services organization). Furthermore, the pilot study suggested that the survey had good levels of relevance, accuracy, and acceptability for GPs, with only one negative answer in three out of four items and none in the other.

A more comprehensive validation of the questionnaire using quantitative methods will be carried out in the context of the EASY-NET program. Within this program, the survey will be administered before and after the implementation of an A&F intervention in order to measure changes in GPs’ knowledge, attitudes, and behaviors. Furthermore, it will evaluate the associations between these domains and the intervention efficacy. The characteristics of GPs that influence the domains of the survey answers and changes after the intervention will be also assessed. In particular, the GP’s age, number of patients, and whether they work in a territorial functional aggregate will be evaluated in order to assess the link between the knowledge, attitudes, and behaviors of GPs and other individual and work-related variables. 

## 5. Conclusions

In conclusion, we used a three-step methodology to develop the survey, starting with a scoping review to cover all possible relevant sub-domains. Due to the lack of specific instruments that assess the knowledge, attitudes, and behaviors of GPs, the survey is a useful tool that can be administrated before and after the implementation of an A&F intervention in order to assess both baseline characteristics and changes after the intervention. Furthermore, the pre-intervention assessment may be useful to tailor the strategy to target specific characteristics. In agreement with the actual evidence and recommendations, it may help to better understand the pathway linking physicians’ individual factors with the effectiveness of different A&F strategies.

## Figures and Tables

**Table 1:** Data extraction.

Author	Year	Target	Domains Indicated in the Study	Knowledge, Attitudes and Behaviors Domains	Scale
Al-Baho	2012	Trainee general practitioners	Knowledge, attitudes	Knowledge, attitudes	Five-point Likert scale
Jensen-Doss	2018	Multidisciplinary mental health team	Attitudes	Attitudes	Five-point Likert scale
Haynes	2019	Residents	Behaviors	Behaviors	Four-point frequency scale
Fahim	2021	Surgeons	Theoretical Framework Domain	Knowledge, attitudes, behaviors	Five-point Likert scale
Stone	2019	Multidisciplinary oncology team	Knowledge, opinions	Knowledge, attitudes	Five-point Likert scale
Taylor	2016	Multidisciplinary oncology team	Barriers	Knowledge, attitudes, behaviors	Five-point Likert scale
Ghaderi	2013	Surgeons	Knowledge, attitudes, behaviors, opinions, barriers	Knowledge, attitudes, behaviors	Five-point Likert scale
Lloyd	2014	Different specialties	Attitudes, barriers	Attitudes, behaviors	Open-ended question
McWilliams	2017	Psychiatry residents	Attitudes, experiences	Attitudes, behaviors	Yes/No—Five-point Likert scale
Bertels	2013	Foundation year one doctors	Views, problems and preferred methods	Attitudes, behaviors	Five-point Likert scale

**Table 2:** Pilot study for assessing the acceptability, relevance and accuracy of the questionnaire.

Question	Score (n = 15)	
	Low (1–3)	High (4–5)
In your opinion, how easy is the questionnaire to answer?	1 6.7%	14 93.3%
In your opinion, how do you rate the readability of the questions?	0 0%	15 100%
In your opinion, how do you score the relevance of the included items?	1 6.7%	14 93.3%
In your opinion, how accurate is the questionnaire?	1 6.7%	14 93.3%

## Data Availability

Data available on request from the authors.

## Supplementary Materials

The following supporting information can be downloaded at: https://www.mdpi.com/article/10.3390/healthcare11091211/s1, Search strategy; Final version of the questionnaire.
