# Supplementary material for: Developing a Questionnaire Evaluating Knowledge, Attitudes and Behaviors on Audit & Feedback among General Practitioners: A Mixed Methods Study
Source: Healthcare (Basel). 2023 Apr 24;11(9):1211. doi: 10.3390/healthcare11091211 (PMC10178397; doi:10.3390/healthcare11091211)
Supplement: Supplementary file 1 [file healthcare-11-01211-s001.zip › healthcare-2329893-supplementary.pdf]

Database: Ovid MEDLINE(R)

Date of search: December 06, 2021

1	(audit* adj3 feedback).tw.	(3680)
2	*Clinical Audit/	(757)
3	*Medical Audit/	(7028)
4	*Management Audit/	(1275)
5	*Benchmarking/	(6012)
6	"Commission on Professional and Hospital Activities"/	(289)
7	*Feedback, Psychological/	(1882)
8	*Peer Review, Health Care/	(828)
9	(audit or audits or auditing).ti.	(15168)
10	feedback.ti.	(26413)
11	1 or 2 or 3 or 4 or 5 or 6 or 7 or 8 or 9 or 10	(54540)
12	Family Practice/	(66219)
13	Professional Competence/	(24949)
14	Clinical Competence/	(100463)
15	Physician's Practice Patterns/	(64857)
16	*Quality Assurance, Health Care/	(32515)
17	*Quality of Health Care/	(39020)
18	((health* personnel or health care personnel or physician? or doctor? or clinician? or provider? or practitioner? or professional? or clinical) adj3 (skill or skills or behaviour or behavior or competence)).tw.	(40362)
19	((clinical or medical or private or general or family or professional) adj practice?).tw.	(302266)
20	(practice pattern? or pattern of practice).tw.	(9553)
21	(quality adj (assurance or improvement or control)).tw.	(113424)
22	(health care quality or healthcare quality or quality of healthcare or quality of health care or quality of care).tw.	(66391)
23	((influenc* or chang*) adj3 (behaviour* or behavior*)).tw.	(95696)
24	12 or 13 or 14 or 15 or 16 or 17 or 18 or 19 or 20 or 21 or 22 or 23	(826234)
25	11 and 24	(9568)
26	attitude*.ti,ab.	(166447)
27	acceptab*.ti,ab.	(201173)
28	point of view.ti,ab.	(45714)
29	(knowledge or barrier* or facilitator* or feasibility).ti,ab.	(1314176)
30	26 or 27 or 28 or 29	(1631448)
31	25 and 30	(1604)
32	limit 31 to yr="2000 -Current"	(1428)

Database: Embase

Date of search: December 06, 2021

1	(audit* adj3 feedback).tw.	(4682)
2	*clinical audit/	(1811)
3	*Medical Audit/	(15575)
4	*benchmarking/	(1806)
5	psychological feedback/	(428)
6	(audit or audits or auditing).ti.	(29960)
7	feedback.ti.	(28394)

8	1 or 2 or 3 or 4 or 5 or 6 or 7	(65460)
9	general practice/	(80450)
10	professional competence/	(33142)
11	*health care quality/	(75116)
12	((health* personnel or health care personnel or physician? or doctor? or clinician? or provider? or practitioner? or professional? or clinical) adj3 (skill or skills or behaviour or behavior or competence)).tw.	(53105)
13	((clinical or medical or private or general or family or professional) adj practice?).tw.	(416705)
14	(practice pattern? or pattern of practice).tw.	(15028)
15	(quality adj (assurance or improvement or control)).tw.	(172787)
16	(health care quality or healthcare quality or quality of healthcare or quality of health care or quality of care).tw.	(89566)
17	((influenc* or chang*) adj3 (behaviour* or behavior*)).tw.	(117092)
18	9 or 10 or 11 or 12 or 13 or 14 or 15 or 16 or 17	(943380)
19	8 and 18	(9197)
20	attitude*.ti,ab.	(206335)
21	acceptab*.ti,ab.	(276134)
22	point of view.ti,ab.	(62063)
23	(knowledge or barrier* or facilitator* or feasibility).ti,ab.	(1661277)
24	20 or 21 or 22 or 23	(2077882)
25	19 and 24	(1641)
26	limit 25 to (human and yr="2000 -Current")	(1370)

Database: APA PsycInfo

Date of search: December 06, 2021

1	(audit* adj3 feedback).tw.	(1923)
2	Clinical Audits/	(551)
3	(audit or audits or auditing).ti.	(1747)
4	feedback.ti.	(14924)
5	1 or 2 or 3 or 4	(18049)
6	exp Family Physicians/ or exp Primary Health Care/	(21016)
7	Professional Competence/	(8320)
8	exp "Quality of Care"/	(14116)
9	((health* personnel or health care personnel or physician? or doctor? or clinician? or provider? or practitioner? or professional? or clinical) adj3 (skill or skills or behaviour or behavior or competence)).tw.	(18626)
10	((clinical or medical or private or general or family or professional) adj practice?).tw.	(76248)
11	(practice pattern? or pattern of practice).tw.	(951)
12	(quality adj (assurance or improvement or control)).tw.	(10277)
13	(health care quality or healthcare quality or quality of healthcare or quality of health care or quality of care).tw.	(18365)
14	((influenc* or chang*) adj3 (behaviour* or behavior*)).tw.	(76149)
15	6 or 7 or 8 or 9 or 10 or 11 or 12 or 13 or 14	(217302)
16	5 and 15	(1381)
17	attitude*.ti,ab.	(215418)
18	acceptab*.ti,ab.	(44313)
19	point of view.ti,ab.	(21431)

20	(knowledge or barrier* or facilitator* or feasibility).ti,ab.	(419696)
21	17 or 18 or 19 or 20	(649374)
22	16 and 21	(293)

Final version of the questionnaire

KNOWLEDGE

1. How do you rate your knowledge on Audit and Feedback?
☐ VERY LOW ☐ LOW ☐ ACCEPTABLE ☐ GOOD ☐ VERY GOOD

2. Do you know the difference between criteria and standard?
☐ YES ☐ NO

3. How do rate your knowledge on process and outcome indicators on chronic disease?
☐ VERY LOW ☐ LOW ☐ ACCEPTABLE ☐ GOOD ☐ VERY GOOD

4. How do rate your competence in defined a standard of care?
☐ VERY LOW ☐ LOW ☐ ACCEPTABLE ☐ GOOD ☐ VERY GOOD

5. How do rate your ability to professional updating through the consultation of clinical guidelines?
☐ VERY LOW ☐ LOW ☐ ACCEPTABLE ☐ GOOD ☐ VERY GOOD

6. Are you aware of programs which systematically disseminate results of process and outcome health care indicators?
☐ YES ☐ NO

7. How do rate your competence in consultation and interpretation of process and outcome indicators at District, Local Health Authority and regional level?
☐ VERY LOW ☐ LOW ☐ ACCEPTABLE ☐ GOOD ☐ VERY GOOD

8. How do rate your competence in consultation and interpretation of data extracted from your patient management software?
☐ VERY LOW ☐ LOW ☐ ACCEPTABLE ☐ GOOD ☐ VERY GOOD

ATTITUDES

9. Do you think A&F is useful to improve your clinical practice?
☐ NOT AT ALL ☐ SLIGHTLY ☐ SOMEWHAT ☐ A LOT ☐ VERY MUCH

10. Do you think it is important to participate in A&F activities?
☐ NOT AT ALL ☐ SLIGHTLY ☐ SOMEWHAT ☐ A LOT ☐ VERY MUCH

11. Do you think that participate in A&F activities is part of your job tasks?
☐ NOT AT ALL ☐ SLIGHTLY ☐ SOMEWHAT ☐ A LOT ☐ VERY MUCH

12. Do you think that participate in A&F activities take away time to your clinical practice?
☐ NOT AT ALL ☐ SLIGHTLY ☐ SOMEWHAT ☐ A LOT ☐ VERY MUCH

13. Do you think that participate in A&F activities have to be rewarded with same sort of incentive?
☐ NOT AT ALL ☐ SLIGHTLY ☐ SOMEWHAT ☐ A LOT ☐ VERY MUCH

14. Do you think it would be important your involvement in the development of A&F activities?
☐ NOT AT ALL ☐ SLIGHTLY ☐ SOMEWHAT ☐ A LOT ☐ VERY MUCH

15. Do you think A&F is useful to improve your patient health?
☐ NOT AT ALL ☐ SLIGHTLY ☐ SOMEWHAT ☐ A LOT ☐ VERY MUCH

16. Do you think that participate in A&F activities may improve the collaboration among healthcare professionals (such as specialists, nurses, service managers)?
☐ NOT AT ALL ☐ SLIGHTLY ☐ SOMEWHAT ☐ A LOT ☐ VERY MUCH

17. If an indicator of your clinical practice did not achieve the standard of care, do you think it will be important to change clinical behaviors (including those under your control)?
☐ NOT AT ALL ☐ SLIGHTLY ☐ SOMEWHAT ☐ A LOT ☐ VERY MUCH

18. Do you think A&F is useful to avoid diagnostic or therapeutic mistakes?
☐ NEVER ☐ RARELY ☐ SOMETIMES ☐ FREQUENTLY ☐ VERY FREQUENTLY

19. Do you think that participate in A&F activities may improve your job satisfaction?
☐ NOT AT ALL ☐ SLIGHTLY ☐ SOMEWHAT ☐ A LOT ☐ VERY MUCH

20. Do you think that receiving feedbacks on your activity may improve your clinical practice?
☐ NOT AT ALL ☐ SLIGHTLY ☐ SOMEWHAT ☐ A LOT ☐ VERY MUCH

21. Do you think that receiving feedbacks on your activity may be useful to monitor your patient health?
☐ NOT AT ALL ☐ SLIGHTLY ☐ SOMEWHAT ☐ A LOT ☐ VERY MUCH

22. Do you think that receiving feedbacks on your activity may add relevant information to your clinical practice?
☐ NOT AT ALL ☐ SLIGHTLY ☐ SOMEWHAT ☐ A LOT ☐ VERY MUCH

23. Do you feel judged by receiving a feedback report on your clinical practice?
☐ NOT AT ALL ☐ SLIGHTLY ☐ SOMEWHAT ☐ A LOT ☐ VERY MUCH

24. Do you think that indicators calculated from administrative data reflect your clinical practice?
☐ NOT AT ALL ☐ SLIGHTLY ☐ SOMEWHAT ☐ A LOT ☐ VERY MUCH

25. Do you think it is important that your performance remain under the threshold standard?
☐ NOT AT ALL ☐ SLIGHTLY ☐ SOMEWHAT ☐ A LOT ☐ VERY MUCH

26. Do you think it is important to compare your indicator results with those of your colleagues?
☐ NOT AT ALL ☐ SLIGHTLY ☐ SOMEWHAT ☐ A LOT ☐ VERY MUCH

27. Do you think it is important to compare your indicator results with the mean values of your functional aggregate/District/Local Health Authority?
☐ NOT AT ALL ☐ SLIGHTLY ☐ SOMEWHAT ☐ A LOT ☐ VERY MUCH

BEHAVIORS

28. Have you ever participated in A&F activities?
☐ YES ☐ NO

29. If yes, did you modify your clinical practice taking into account the critical issues emerged?
☐ NOT AT ALL ☐ SLIGHTLY ☐ SOMEWHAT ☐ A LOT ☐ VERY MUCH

30. Have you ever received from your Local Health Authority a report of your clinical practice?
☐ NEVER ☐ RARELY ☐ SOMETIMES ☐ FREQUENTLY ☐ VERY FREQUENTLY

31. Have you ever consulted process or outcome healthcare indicators disseminated at national or local level?
☐ NEVER ☐ RARELY ☐ SOMETIMES ☐ FREQUENTLY ☐ VERY FREQUENTLY

32. Have you ever used your management software to get synthesis data or to monitor your clinical practice and your patient health?
☐ NEVER ☐ RARELY ☐ SOMETIMES ☐ FREQUENTLY ☐ VERY FREQUENTLY

33. Have you ever compared your indicator results with those of your colleagues?
☐ NEVER ☐ RARELY ☐ SOMETIMES ☐ FREQUENTLY ☐ VERY FREQUENTLY

34. Have you ever compared your indicator results with the mean values of your functional aggregates/District/Local Health Authority?
☐ NEVER ☐ RARELY ☐ SOMETIMES ☐ FREQUENTLY ☐ VERY FREQUENTLY

35. Have you ever modified your clinical practice after the consultation of a synthesis report of your activity?
☐ NEVER ☐ RARELY ☐ SOMETIMES ☐ FREQUENTLY ☐ VERY FREQUENTLY

36. To your knowledge, in your functional aggregate are organized structured meetings aiming to compare clinical and organizational results?
☐ NEVER ☐ RARELY ☐ SOMETIMES ☐ FREQUENTLY ☐ VERY FREQUENTLY
